# Traumatic dental injuries: Prevalence and associated risk factors among school children aged 12-15 years in Western Uttar Pradesh, India

**DOI:** 10.6026/973206300212857

**Published:** 2025-08-31

**Authors:** Mukund Sharma, Vikas Singh, Pradeep Tangade, Ankita Jain, Garima Rani, Prajwal Singh Tomar

**Affiliations:** 1 Department of Public Health Dentistry at Teerthanker Mahaveer Dental College and Research Centre, Moradabad, Uttar Pradesh, India; 2 Department of Public Health Dentistry at Maharishi Markandeshwar College of Dental, Science & Research, Mullana-Ambala, Haryana, India; 3 Department of Periodontology at Shree Bankey Bihari Dental College & Research Centre, Ghaziabad, Uttar Pradesh, India

**Keywords:** Trauma, dental injury, oral health, etiology, awareness

## Abstract

Traumatic dental injures shows a serious impact on oral health and its incidences are increasing significantly over time. Permanent anterior teeth commonly affected. Study focused on prevalence of dental trauma among 12-15 years old school going children of Ghaziabad and assess risk factors related to traumatic injuries. A cross sectional study carried out by using a structured questionnaire and clinical examination to collect data from 323 subjects with a history of dental injury. Results revealed that prevalence of traumatic injuries found higher and on the whole, it was more in girls when compared to boys. Most common etiology of trauma being inability to recollects the incident. There is a need to enhance the understanding and awareness of the prevention of traumatic injuries.

## Background:

The presence of a condition that has a significant impact on individuals or leads to considerable social costs, while at the same time being preventable, warrants recognition as a public health issue. Traumatic dental injuries (TDIs) fulfil these criteria [1]. Globally, TDIs represent a significant health burden and are considered a serious social problem, particularly in pediatric and adolescent populations [2]. Most dental injuries affect the anterior teeth, which are both functionally and esthetically important [3]. Epidemiological studies predict that dental trauma may become more prevalent than dental caries and periodontal disease in youth, highlighting its growing importance in preventive dentistry. In early childhood, dental injuries are relatively uncommon; however, as children mature, they often become more impulsive and inattentive, increasing their susceptibility to accidents [4]. The rise in physical activity levels among children has also been directly associated with an increase in the incidence of dental trauma. The consequences of TDIs extend beyond clinical complications and can significantly affect overall quality of life. Social, psychological and economic impacts of dental trauma have been extensively documented, demonstrating its far-reaching public health implications [5, 6]. Considering that TDIs among children aged 12-15 years remain a major unresolved concern in many communities, there is a need to evaluate their incidence and identify associated risk factors to raise awareness and provide valuable preventive insights. Therefore, it is of interest to report the incidence and risk determinants of TDIs in this age group.

## Materials and Methods:

A cross sectional study performed on school going students of twelve to fifteen years, on both sex who regularly attend school in Ghaziabad Uttar Pradesh, India. Ghaziabad city was separated by five parts- center, east, west, north & south part as per location, schools chosen. A list of schools was created in the first stage, complete with addresses, phone numbers, class counts and total number of student enrolled. Schools were separated into five areas in the second stage and ten schools were chosen randomly in the third stage. Each school's estimated student population was chosen evenly. To examine permanent dentition and surrounding structures of oral cavity, Classification of TDIs (code according WHO) was used, containing injuries of teeth, supporting units, gingival and oral mucosa. Collection of data was done from 323 school going children aged 12 to 15 years, using a pre-validated structured Questionnaire and clinical examination, students with clinical findings of oral injury, interviewed and examined. ADA type three techniques were applied for examination by using standard mouth mirrors and probes under proper light. Measures for Proper infection control were taken. Age, gender, Etiology of injury, Place of occurrence, Tooth region involved, type of trauma, over-jet of incisor and coverage of lips recorded. To determine degree of over-jet (dichotomise to ≤ 3 mm and > 3 mm), as per the guidelines from 2013 WHO Basic Oral Health Survey, Community Periodontal Index (CPI) probe was used. By direct facial examine, competent or incompetent lips seal were determined. Before beginning, Permission from ethical committee of Teerthanker Mahaveer Dental College and Research Centre was obtained with reference number of TMDCRC/IEC/TH/22-23/PHD 03. An informed consent obtained from the participants and institutions who willingly participated in the study.

## Inclusion criteria:

Children who are available and willing to take part in survey and under age group of 12-15 years

## Exclusion criteria:

[1] Widespread caries in Permanent dentition.

[2] Dental deformities like amelogenesis/dentinogenesis imperfecta, enamel/dentin hypocalcification.

[3] Subjects with orthodontic treatment.

[4] Subjects with major systemic illness.

[5] Subjects not willing to take part or absent during survey.

## Study sample:

The sample size calculated 323 by using following formula:

n= [Z2 x P x (1-P)]/e2

Where,

n= Sample Size

Z = values for standard normal distribution corresponding desired confidence level (Z=1.96 for 95% CI)

P = expected true proportion (30%)

e = desired precision (half desired CI width).

## Statistical analysis:

Data were collected through the survey and entered into Microsoft Excel sheets. Statistical analysis was performed using SPSS version 22, with the level of significance set at p < 0.05.

## Results:

The age distribution of participants, shows that 35.0% were 12 years old (113 participants), 25.1% were 13 years old (81 participants), another 25.1% were 14 years old (81 participants) and 14.9% were 15 years old (48 participants). The mean and standard deviation is 13.20 ± 1.077, indicating that the ages are moderately dispersed around the average. Of the total participants, 37.8% (122) were male and 62.2% (201) were female, indicating a higher proportion of females in the study group. The mean gender value is 1.62 with standard deviation of 0.486. Overjet distribution among total participants, shows that 55.1% (178 participants) had an overjet of 3 mm or less, while 44.9% (145 participants) had an overjet greater than 3 mm with mean overjet value 1.45 & standard deviation 0.498 (Table 1, 2 and 3 - see PDF). Distribution of lip coverage among total participants, with 62.8% (203 participants) had competent lip coverage and 37.2% (120 participants) had incompetent lip coverage with mean value 1.37 & standard deviation 0.484. Most common cause as inability to recollect the incident (40.6%, 131 participants), followed by falls (29.7%, 96 participants) and biting hard food (15.8%, 51 participants). Other reported causes include impacts (7.1%, 23 participants), sports activities (4.3%, 14 participants), road accidents (1.5%, 5 participants) and violence (0.9%, 3 participants). Mean etiology value 4.22 & standard deviation 2.455, indicating variation in the reported causes of trauma. The most common locations were home (35.0%, 113 participants) and school (29.4%, 95 participants). Other reported locations include playgrounds (13.6%, 44 participants), roadsides (9.9%, 32 participants) and other unspecified places (12.1%, 39 participants). Mean occurrence value 2.40 & standard deviation 1.325, reflecting variability in the reported locations of trauma. The upper anterior region was most frequently affected (59.8%, 193 participants), followed by the lower anterior region (35.3%, 114 participants). Less commonly affected regions include the lower posterior (4.3%, 14 participants) and upper posterior (0.6%, 2 participants). Mean value 1.84 & standard deviation 1.050, indicating variability in affected regions.

## Discussion:

TDIs are present all throughout the world and are highly prevalent in all dentition. Gender and age are likely less important determinants of TDIs than an individual's activities and surroundings. It is required to offer a risk profile explaining why certain individuals experience multiple dental trauma episodes. TDI trend appears to be constant, with variations primarily reflecting local differences [7]. Like presented study, Paiva *et al.* also showed that 12-year-old school children had a higher prevalence of TDI. Exaggerated overjet was linked to the occurrence of TDI, although gender, socioeconomic position and lip protection did not influence the results. TDI and the socioeconomic indicator under analysis did not correlate [8]. As per Traebert *et al.* [9], enamel fractures were the most common injury type discovered, which are similar in the presented study. Just 27.6% of the 87 traumatized teeth received treatment. The most often applied treatment was restoration. The majority of TDI instances (17.8%) happened at home, while 17.8% happened at school, which is also similarly reflected in our study, TDI instances at home (35%) followed by school (29.4%). The primary activities associated with TDI aetiology were striking, but this finding isn't similar to our study where it was mentioned that most of the participants can't recollect the cause of trauma; more injuries were seen in 12-year-old subjects, given vulnerability to demographic changes during the transition from childhood to adulthood [10]. In a survey presented by Stockwell [11], compared to boys (11.1%), girls (12.8%) experienced a substantially higher amount of trauma. This is in favor of the present study where girls (62.2%) relatively experienced more traumatic dental injuries than boys (37.8%) of total subjects. According to David *et al.* [12], incidence of front teeth trauma was 6%, while the present study also revealed a high number of trauma to front teeth (95.1% upper & lower combined). As per David *et al.* the most often impacted teeth were right central incisors. Compared to other research, incidence of damaged front teeth among 12-year-olds was low. In a survey, incidence of oral trauma was recorded as 6.19%, showing that non-complicated coronal fracture was the most common type. Similar to other overall and oral health issues, oral injury prevention is seen as more important from all angles than therapy. Thus, it is necessary to start community education initiatives about the causes, prevention and cure of oral injuries [13]. Folakemi [14] described in his study that few caregivers sought dental care for their wards right away after trauma and most injuries happened at home. In order to prevent complications and complicated treatments, parents and educators are encouraged to seek care for traumatic oral injury as soon as possible. In the present study, with mean gender value 1.62 and standard deviation of 0.486, it was observed that women were more impacted than men overall. This contradicts the findings of other researchers who show that injuries are more common in men. One possible explanation for this discrepancy is that women are now increasingly participating in activities that increase the risk of tooth trauma [15, 16-17]. From the survey conducted by Goswami *et al.* [18], it was noted that soft tissue injuries were high (50.6%) among their study population, while in the present survey injuries to the gingiva or oral mucosa were found relatively very low (approximately 1%). This huge difference could be due to the difference between study areas of both studies, as Goswami *et al.* took their sample from a hospital and the present research was done in schools. Traumatic dental injury (TDI) in children and adolescents has become one of the most serious dental public health problem, and the prevalence of dental injuries in Uttar Pradesh was high and it has a great potential to be considered as an emerging public health problem [19, 20].

## Conclusion:

Traumatic dental injuries among children remain a significant concern, with higher prevalence observed in girls due to increased participation in diverse activities. Lack of awareness and delayed response from children and caregivers highlight gaps in dental health knowledge. Strengthening education, prevention and timely care is essential to reduce the burden and long-term consequences of dental trauma.

## Limitations:

Even after all the findings of present study there could be some limitations which should be addressed,

[1] The study targeted on a specific population, which is 12-15 year old school going children, this makes the study limited under a specific group. For further researches another age group could be opted for more findings.

[2] There might be chances of response bias by the students under the influence of other's response or improper knowledge.

[3] Gender wise distribution might be affected due to the availability or willingness to take part in study.

[4] Institutional environment or their arrangements during the time of survey also affects the efficiency of findings.

[5] More technology can be utilising to improve the future researches.

## Recommendations:

These are the recommendation as per the findings:-

[1] Integration of dental health education in School's curriculum.

[2] Integration of basic Dental treatment availability in the school campus for regular check-ups.

[3] Interview and Discussion with teachers and parents or caretaker to increase awareness.

[4] To make children understand about the importance of oral health and also to be alert while performing any activity which is a risk factor for dental injuries.

[5] By Providing a safe platforms and environment to young children these injuries can also be controlled.

[6] Create a fear free opinion about dental treatment for children so that they can cooperate in getting treatment and also become confident about it.

## References

[1] Nagarajappa R (2020). Journal of dental sciences..

[2] Azami-Aghdash S (2015). Medical journal of the Islamic Republic of Iran..

[3] Juneja P (2018). Clujul medical..

[4] Sari ME (2014). Nigerian journal of clinical practice..

[5] Born CD (2019). Clinical and experimental dental research..

[6] Pagadala S, Tadikonda DC. (2015). International Archives of Integrated Medicine..

[7] Glendor UL. (2008). Dental traumatology..

[8] Paiva PC (2015). Ciência & Saúde Coletiva..

[9] Traebert J (2006). Dental traumatology..

[10] Paiva PC (2015). International journal of paediatric dentistry..

[11] Stockwell AJ. (1988). Community dent oral epidemiol..

[12] David J (2009). Dental traumatology..

[13] Faus-Damiá M (2011). Med Oral Patol Oral Cir Bucal..

[14] https://www.researchgate.net/.

[15] Huang B (2009). Dent Traumatol..

[16] Malikaew P (2006). Community Dent Health..

[17] Wendt FP (2010). Dent Traumatol..

[18] Goswami M, Aggarwal T. (2021). International journal of clinical pediatric dentistry..

[19] Chopra A (2014). Arch Trauma Res..

[20] Gupta R (2021). J Indian Assoc Public Health Dent..

